# The Effect of the Pre-Existing VC on the Evolution of Precipitate and Mechanical Properties in the H13 Steel

**DOI:** 10.3390/ma15113970

**Published:** 2022-06-02

**Authors:** Kefei Shi, Fei Zhao, Yuan Liu, Sheng Yin, Ronggui Yang

**Affiliations:** 1 College of Materials and Metallurgy, Guizhou University, Guiyang 550025, China; guskf2477@163.com (K.S.); liuyuan0851@163.com (Y.L.); yinsheng111@163.com (S.Y.); yronggui97@163.com (R.Y.); 2 Key Laboratory for Materials Structure and Strength of Guizhou Province, Guizhou University, Huaxi District, Guiyang 550025, China

**Keywords:** H13 steel, intercritical annealing, carbide, precipitation, high temperature

## Abstract

To further improve the mechanical properties of H13 steel at room and high temperatures, its precipitates were regulated based on the Thermo-Calc results. Scanning electron microscope (SEM), electron backscattering diffraction (EBSD), transmission electron microscope (TEM), and X-ray diffraction (XRD) Rietveld refinement were used to study the effect of the intercritical annealing on the microstructure and mechanical properties of H13 steel. The results show that the intercritical annealing at 850~95 °C increased the VC volume fraction from 2.23 to 3.03~3.48%. Increasing the VC volume fraction could inhibit the M_7_C_3_ precipitation from 10.01 to 6.63~5.72% during tempering. A large amount of VC also promoted the M_23_C_6_ precipitation during tempering at higher dislocation densities. The intercortical annealing simultaneously increased the elongation of H13 steel. An excellent combination (room temperature: ultimate tensile strength (UTS) of 898 MPa and total elongation (TEL) of 19.35%, 650 °C: UTS of 439 MPa, and TEL of 27.80%) could be obtained when intercritical annealing is performed at 900 °C. Meanwhile, after aging at 650 °C for 128 h, the room temperature UTS and TEL decreased by only 31 MPa and 0.52%, respectively.

## 1. Introduction

H13 steels are widely used in forging die, hot extrusion die, and die casting die for their relatively low cost and suitable combinations of strength and appropriate toughness [1,2,3]. At the same time, because of their excellent high-temperature stability, H13 steels have the potential to replace the high production cost of nickel-based superalloy and can be used as high-temperature structural materials such as aircraft engines and high-temperature tubing support parts [4,5,6]. In addition, compared with the development of high-performance and low-cost new materials, adjusting the heat treatment process of H13 to meet the performance requirements, the UTS at room temperature and 650 °C is higher than 890 MPa and 297 MPa, and EL is greater than 19% and 25%, respectively. High-temperature structural support materials will undoubtedly greatly reduce the research and development costs and production costs [7,8], which are also the focus of this paper.

Due to the high content of carbide-forming elements such as Cr, Mo, and V, controlling the phase, size, quantity, and distribution of carbides has become a key factor affecting their performance. Studies have shown that carbides in H13 steel are mainly: Cr-rich and Fe-rich M_23_C_6_, Cr-rich M_7_C_3_, Fe-rich M_3_C, Mo-rich M_2_C, and V-rich MC [9,10,11]. MC and M_2_C phases with simple lattice structures are small and have decomposition temperatures above 1000 °C. Their stability is much higher than those of M3C, M7C3, and M23C6 with complex lattice structures. Therefore, the content, size, and distribution have a positive significance for the high-temperature properties of H13 steel [12].

Many scholars have studied how to increase the content of MC and M_2_C phases in steel. Zhao et al. [13] conducted an isothermal pretreatment at 950 °C for 1 h before the nitriding treatment of H13 steel which effectively increased the VC content in the matrix. Kwon et al. [7] changed the annealing process of the high manganese steel containing 0.5 wt.% V, which extended the annealing time and reduced the cooling rate, thus significantly improving the VC volume fraction. In addition, interphase precipitation is also an essential factor that affects the precipitation behavior of the MC phase. Zhang et al. [14,15,16,17] comprehensively analyzed the effects of the α/γ interface orientation relationship, V content, and phase transition temperature on the precipitation of the VC interphase. They found that VC is more inclined to precipitate at the α/γ interface with high-energy non-Kurdjumov-Sachs (K-S)orientation relationship. The interphase precipitation of VC can be effectively promoted by reducing the phase transition temperature and increasing the V content. It can be seen that the methods to promote the precipitation of MC and M_2_C phases include: (1) the heat treatment at the most favorable thermodynamic temperature for the precipitation of MC and M_2_C phases; and (2) increasing the ratio of the α/γ interface in non-K-S orientation relationship.

At present, the effect of critical region annealing on the precipitation behavior of the MC phase is mainly concentrated in micro-alloyed low-carbon steel [18,19,20], but in medium carbon steel, it was concentrated with complex precipitation [13]. In addition, the effect of the increase in the volume precipitation of MC phases on the M7C3, M23C6 precipitation behavior during the tempering process still needs further studying.

In this work, the effect of the intercritical annealing temperature on the precipitation behavior of carbide as well as the interrelationship between the evolved microstructure and mechanical properties were analyzed.

## 2. Materials and Methods

**Figure 1:**
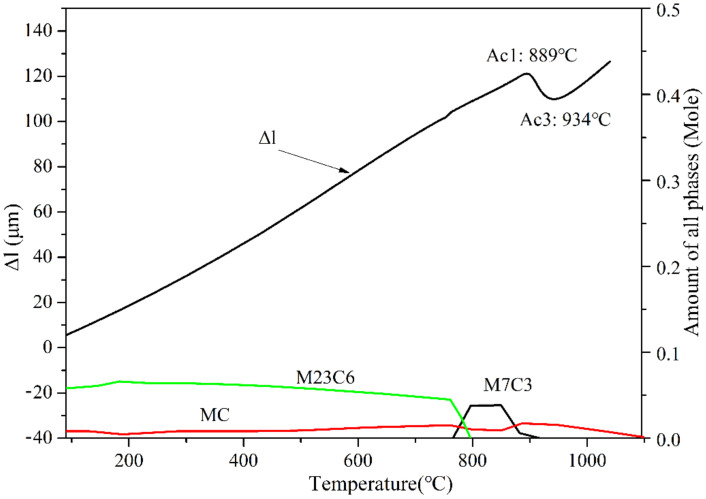
The expansion curve of H13 steel and the equilibrium precipitation curve of the precipitates.

The determined chemical composition of the H13 steel using spectrometers (Bruker, TASMAN Q4-130) is given in Table 1. Figure 1 shows the expansion curve of H13 steel obtained by the phase change meter and the equilibrium precipitation curves of M_23_C_6_, M_7_C_3_, and VC calculated by Thermo-Calc. It is seen from the curve that the phase transformation temperatures of the H13 steel are 889 and 934 °C, respectively, while the precipitation volume of the MC reaches a maximum at 900 ± 50 °C in equilibrium.

**Figure 2:**
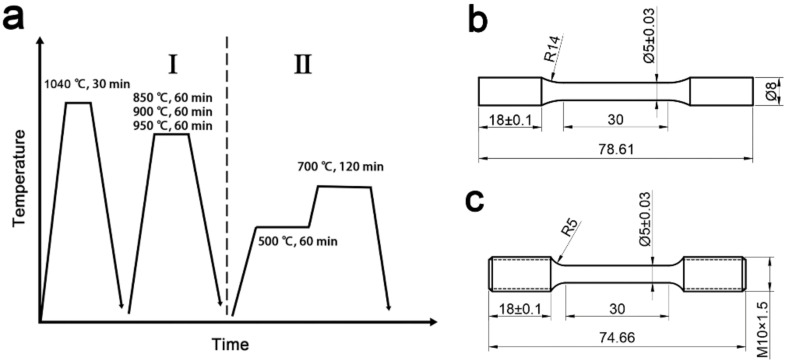
The schematic diagrams of (**a**) heat treatment, (**b**) room temperature, and (**c**) high-temperature tension size (mm).

The heat treatment process is shown in Figure 2. Stage I of the heat treatment was austenitizing at 1040 °C for 30 min and oil quenching. Then, intercritical annealing was performed at 850, 900, and 950 °C for 1 h, and subsequently, oil quenched. This was marked as 850-I, 900-I, and 950-I, respectively. The 0-I was not annealed in order to promote the secondary precipitation of the precipitate and obtain a stable structure at 650 °C. In stage II, the tempering was performed at 500 °C for 60 min, and at 700 °C for 120 min (marked as 850-II, 900-II, 950-II, 0-II) and subsequently oil quenched. The thermal stability test was carried out in a pit furnace at a holding temperature of 650 °C for 128 h. In order to avoid the influence of decarburization, the surface layer of the sample needs to be excised by approximately 1 mm.

The dimensions of the mechanical performance test sample are shown in Figure 2b. These tests of room and high temperatures were performed using the MTS electro-hydraulic servo universal testing machine at a tensile speed of 1 mm/min. It is worth mentioning that before the high-temperature tensile test is carried out, the sample should be kept at test temperature for 10 min. For accuracy purposes, three samples were tested, and the average value was taken to reduce the error.

After grounding and polishing the samples, 4% nitric acid was used for corrosion. Scanning electron microscopy (Zeiss, SUPRA-40, Oberkochen, Germany) was used to observe the microstructure and count the precipitates’ size. From three to five fields of view were selected for size statistics. A transmission electron microscope (FEI Talos F200X) was used to analyze the microstructure and determine the crystal structure of the precipitates. Since the content of the precipitate in the sample was small, the samples were extracted through the electrolytic matrix (the electrolyte was 3 wt.% FeSO_4_, 1 wt.% NaCl, and 0.25 wt.% potassium sodium tartrate, while the current was 0.025 A/cm^2^). The obtained product was washed with 1 mol/L dilute hydrochloric acid, centrifuged, vacuum dried, ground, and then detected by X-ray diffractometer (Bruker D8, Cu target, 40 Kv, 40 mA, continuous scanning, 20~95°, 0.8°/min). Finally, the Fullprof software was used to carry out Rietveld refinement and a quantitative analysis of the precipitate [21].

Assuming that all carbides are spherical, their average diameter could be calculated by image processing software. The improved McCall–Boyd formula was used to calculate the volume fraction of the precipitate, as shown in Equation (1) [22,23]:

(1) $$V_{f}=1.4\pi ND^{2}/6A$$

where *D* is the average size of the precipitates (nm) and *N* is the number of carbides in the statistical area *A* (nm^2^).

## 3. Results and Discussion

### 3.1. Analysis of the Precipitates

**Figure 3:**
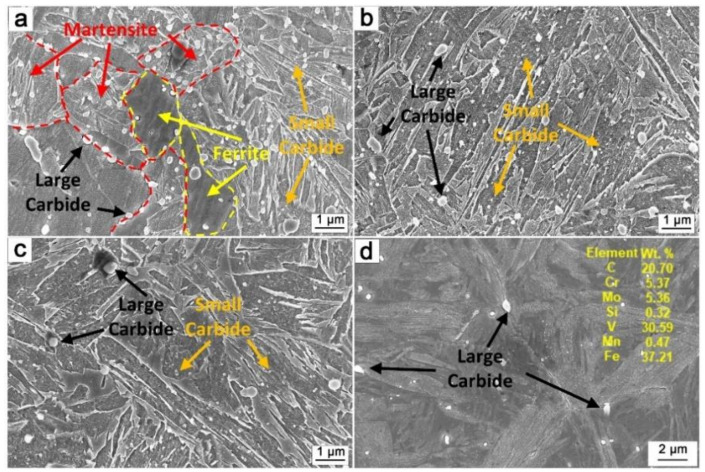
SEM images of stage I: (**a**) 850-I; (**b**) 900-I; (**c**) 950-I; (**d**) 0-I.

**Figure 4:**
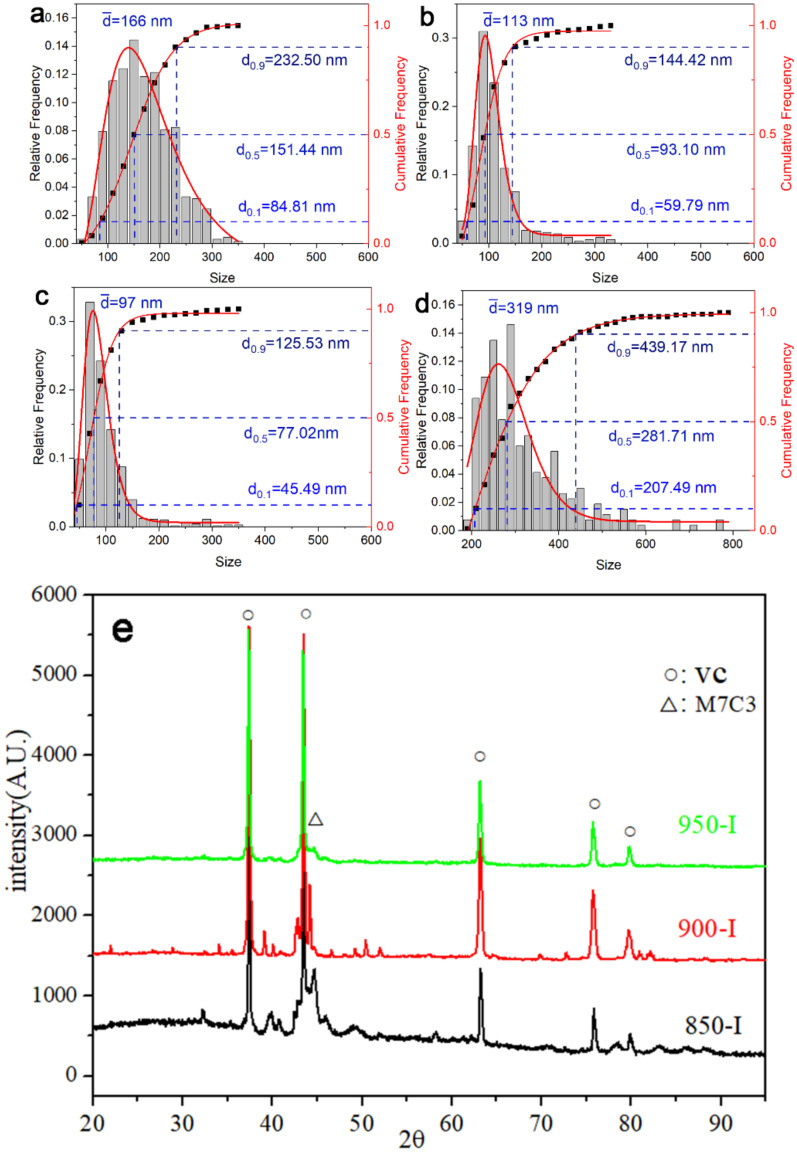
The precipitate sizes and XRD of the first stage I: (**a**) 850-I; (**b**) 900-I; (**c**) 950-I; (**d**) 0-I; and (**e**) XRD of 850-I, 900-I, 950-I.

Figure 3 shows the SEM images of stage I. Figure 3a shows that there is a reverse transformation from martensite to austenite when the H13 steel was annealed at 850 °C for 1 h; however, the reverse austenitic transformation was incomplete and the structure was equiaxed ferrite and secondary martensite. The transformation was complete with the structure of secondary martensite after intercritical annealing at 900 and 950 °C for 1 h (Figure 3b,c). The size of the precipitates reached 166 nm after annealing at 850 °C, while the sizes of those annealed at 900 and 950 °C were only 113 and 97 nm, respectively. The precipitate types in the intercritical annealing sample were VC and M_7_C_3_ phases as qualitatively determined from XRD results (Figure 4). The M_7_C_3_ content gradually increased by decreasing the annealing temperature. The precipitate of 0-I was determined to be mainly VC as detected through EDS analysis.

In the early stage of intercritical annealing, the reverse austenite formation process included nucleation and growth at the interface. Martensite plates were decomposed, resulting in the formation of ferrite. Carbide precipitation behaviors are different in ferrite and austenite due to the difference in the chemical composition induced by the element partitioning during intercritical annealing. C and Mn tended to diffuse into the reversed austenite, while Cr and V tended to be in the ferrite [24]. On the one hand, the M_7_C_3_ nucleation driving force is insufficient with low C content, and a large number of precipitates will not be precipitated in the ferrite (Figure 3a). On the other hand, the increase in the number of γ/α interface promotes the formation of interfacial precipitates which enter the interior of the γ phase from the interface with its growth. When the annealing temperature reaches 850 °C, a two-phase region, a stable γ/α interface is found. The interface will become a diffusion channel for solute atoms promoting the growth of precipitates formed on the interface. Annealing at 900 and 950 °C produces an austenite single-phase region, while the γ/α interface only exists in the early stage of phase transformation. The precipitated VC at the interface does not undergo significant coarsening due to the disappearance of the interface and the local diffusion of V atoms. In addition, abundant C atoms in austenite are conducive to VC nucleation during intercritical annealing. This further reduces the V concentration and suppresses the coarsening behavior. The large-sized VC, which was the undissolved carbides during the austenitizing, was observed in martensite (Figure 3d).

**Figure 5:**
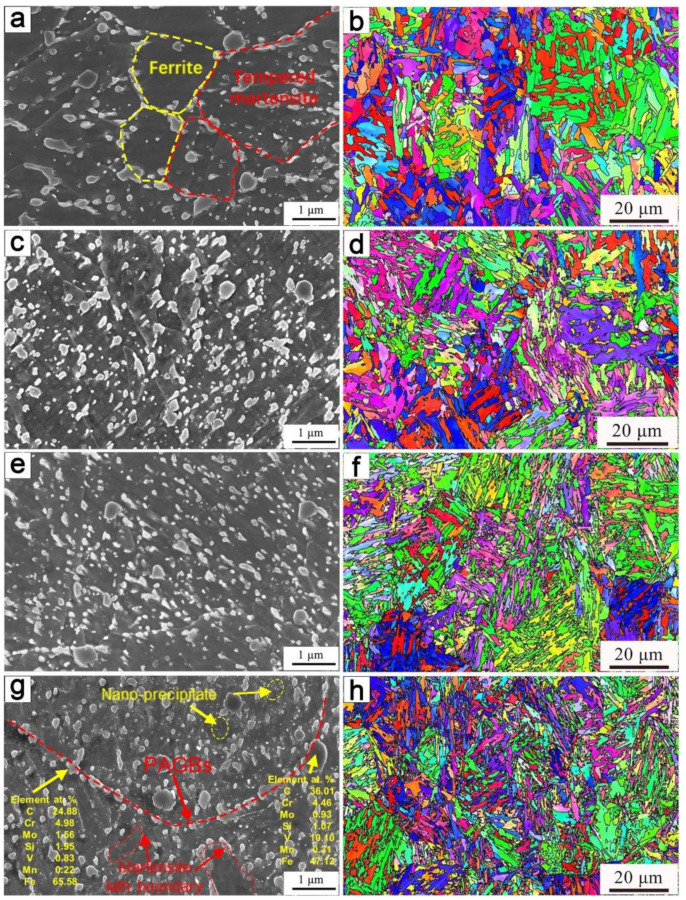
SEM and EBSD images of stage II: (**a**,**b**) 850-II; (**c**,**d**) 900-II; (**e**,**f**) 950-II; (**g**,**h**) 0-II.

Figure 5 observes the microstructure after the two-step tempering. In Figure 5a, the precipitates in ferrite and tempered martensite are different. There are almost no precipitated phases in ferrite due to the low C content and dislocation density. Martensite has a high dislocation density which can promote the precipitation of carbides during tempering so there are a large number of fine precipitates in the martensite. Large-size precipitates were observed along the prior austenite grain boundaries (PAGBs) and martensite lath boundary in Figure 5g. EDS results showed that the precipitates were mainly M_7_C_3_ and M_23_C_6_ phases. At the same time, there are also nano-precipitates with very small sizes between the large-size precipitates. The average size and volume fraction of the precipitates are summarized in Table 2. Annealing at 900 °C reduced the size and volume fraction of the precipitate. Compared with 0-II, the average size of the precipitates decreased by 29 nm, while the volume fraction decreased by ≈ 2.58%.

The substructure size and geometrically necessary dislocation density (ρ^GND^) results obtained by EBSD are summarized in Table 2. The substructure size of the 0-II sample was the smallest—only 1.33 μm. The width of 850-II was the largest, which was 1.98 μm while the size of 950-II was smaller than those of 900-II and 850-II, which was only 1.45 μm. However, fine precipitates and reverse austenite transformation can inhibit the process of grain growth. Therefore, the substructure size of the 950-II is smaller than that of both 850-II and 900-II.

The variation trend of the dislocation density after tempering is related to the content of the secondary martensite and dislocation density before tempering. The secondary martensite content and dislocation density are related to the reverse austenite transformation. With the increase in the critical annealing temperature, on one hand, the transformation of the reversed austenite is complete and the matrix is all secondary martensite. On the other hand, the precipitation of the M_7_C_3_ phase in the matrix gradually decreases, the content of alloying elements in the reverse transformation of austenite increases, and the dislocation density of the secondary martensite increases. Therefore, the maximum dislocation density is obtained in 0-II, while the minimum dislocation density is obtained in 850-II.

**Figure 6:**
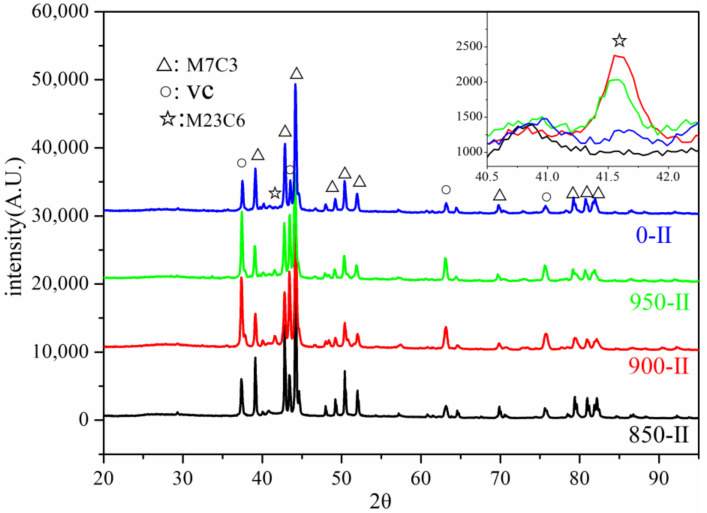
XRD patterns of electrolytic extraction products in stage II.

The M_7_C_3_ phase precipitation significantly increased after tempering (Figure 6). In addition, the stage’s II XRD patterns revealed that a small amount of M_23_C_6_ precipitated in other processes except for 850-II. Table 2 shows the content of each phase obtained by Rietveld refinement using Fullprof software. The results of the table show that the content of VC increased while the content of M_7_C_3_ slightly decreased after the intercritical annealing. Compared with 0-II, the VC contents of 850-II and 900-II increased from 2.23 to 3.03 and 3.05%, respectively, while that of 950-II increased to 3.48%. Compared with C-II, the M_7_C_3_ content of 850-II, 900-II and 950-II dropped from 10 to approximately 6%. This was because of the precipitation of a large amount of VC after annealing that consumed the C element, reduced the C concentration in the matrix, and inhibited the nucleation and growth of M_7_C_3_ to a certain extent.

**Figure 7:**
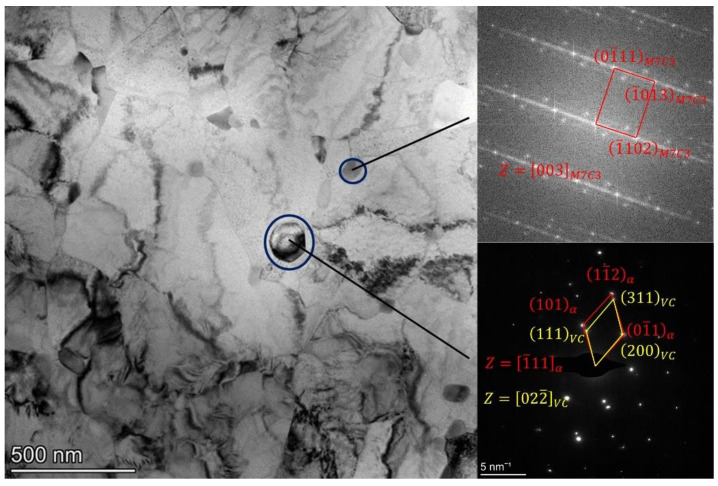
The precipitation and the selected area electron diffraction pattern of 900-II.

**Figure 8:**
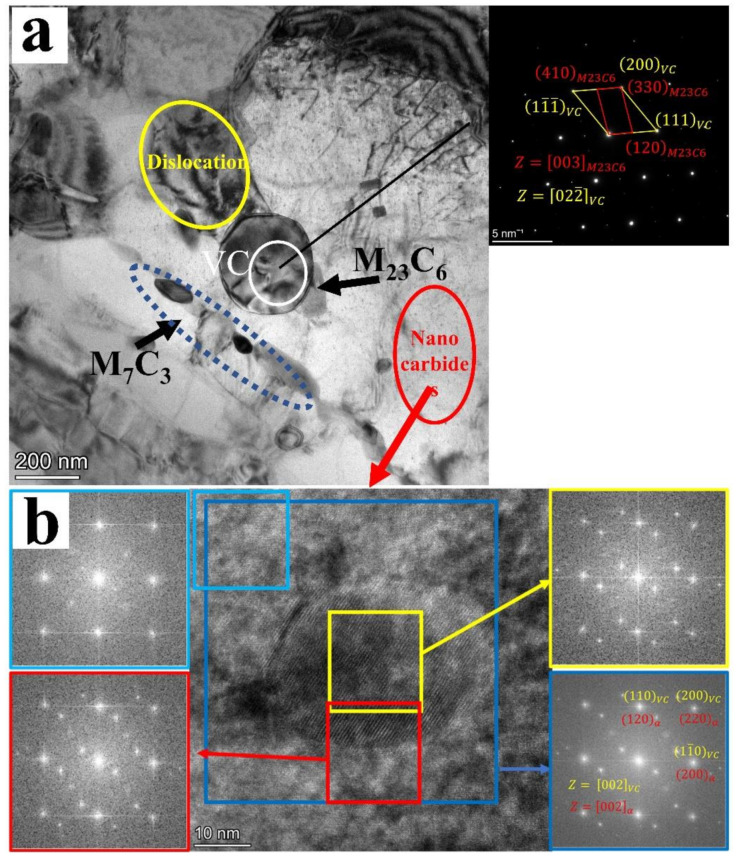
(**a**) Distribution of the precipitated phase in the tempered martensite; (**b**) High-resolution and Fourier change images of the selected area of nano VC in 900-II.

The precipitate evolution of H13 steel during intercritical annealing and the microstructures tested of 900-II were examined using the TEM. Figure 7 and Figure 8 represent the TEM images of 900-II. The orientation relationship between carbides (M_23_C_6_, M_7_C_3_, and VC) and the matrix can be obtained by analyzing the diffraction spots of the selected area and the Fourier change of the high-resolution image. As an essential strengthening phase in steel, the MC phase is mainly a spherical face-centered cubic (FCC) structure. Generally speaking, when the MC-type precipitate of the FCC structure is precipitated in the matrix of the body-centered cubic (BCC) structure, the orientation relationship between the two becomes in line with the Baker–Nutting orientation relationship [25,26] ${\left(100\right)}_{MC}∥{\left(100\right)}_{\alpha }$, ${\left[010\right]}_{MC}∥{\left[110\right]}_{\alpha }$. The small size (<50 nm) of the VC and BCC matrix diffraction spots completely overlap, namely: ${\left(100\right)}_{VC}∥{\left(110\right)}_{\alpha }$, ${\left[001\right]}_{VC}∥{\left[001\right]}_{\alpha }$, as shown in Figure 8b. This is a variant of the Baker–Nutting orientation relationship [27,28]. However, the orientation relationship between the larger VC and the matrix underwent certain change. The ${\left(100\right)}_{VC}$ and the ${\left(110\right)}_{\alpha }$ crystal planes no longer remained parallel, but a deflection of approximately 1.60° appeared (Figure 7). This was because the VC growth was accompanied by the enrichment of other elements such as Mo. These elements would change the lattice parameters of the carbides, which in turn causes the crystal orientation to change. On the other hand, it is also related to VC formation, where VC of small size (<50 nm) is mainly precipitated in situ in the ferrite matrix during the tempering stage. At this time, the degree of mismatch between the precipitate and the matrix is small, the growth of the driving force is insufficient, and an excellent semi-coherent interface can be maintained, as manifested in the BN orientation relationship. In this experiment, large-size VC precipitates were produced during both quenching and intercritical annealing (Figure 4). In the early stage of intercritical annealing, a large amount of VC nucleated at the γ/α interface with more considerable orientation difference. As the intercritical annealing progressed, the γ phase interface would migrate to the α phase, and the pre-existing VC of the γ/α interface goes to the γ phase. After quenching, the γ phase transformed into the martensite phase. The VC and the matrix were not precipitated in situ. Thus, the orientation did not strictly satisfy the BN orientation relationship.

In Figure 8a, the elliptical M_7_C_3_ phase is precipitated along the interface while the rectangular M_23_C_6_ phase is attached to the VC nucleus. The diffraction spots show that there was an orientation relationship between M_23_C_6_ and VC represented in ${\left(200\right)}_{\mathrm{VC}}//{\left(330\right)}_{M_{23}C_{6}}$ ${\left[02\bar{2}\right]}_{\mathrm{VC}}//{\left[009\right]}_{M_{23}C_{6}}$. Previous studies have shown that there is a certain probability that the MC phase can become the core of the heterogeneous nucleation of M_23_C_6_ [29,30,31]. The (111) crystal plane of the face-centered cubic VC has atomic accumulation to become the M_23_C_6′_s nucleation point. Then, M_23_C_6_ grows towards the matrix along with the (120) (the short side of the M_23_C_6_) and (110) (the long side of the M_23_C_6_) crystal planes, which is consistent with the results in this paper.

In this experiment, different annealing temperatures resulted in entirely different M_23_C_6_ contents which are related to the pre-existing VC content and the dislocation density before tempering. On the one hand, the existence of a large amount of pre-stored VC can be used as the nucleation point of M_23_C_6,_ which is advantageous for promoting its precipitation. It can be observed in Figure 3 and Figure 4 that the VC content of the samples that had undergone intercritical annealing before tempering was much higher than that of the samples without intercritical annealing. These pre-existing VCs had a large size and a significant degree of mismatch with the matrix, providing conditions for the nucleation of M_23_C_6_. On the other side, the high dislocation density could provide the driving force for the precipitation of M_23_C_6_ during the tempering process. Table 2 shows the geometrically necessary dislocation density after tempering. It can be observed that since the reverse austenitic transformation was incomplete, the dislocation density was lower than in other processes for 850-II. Although there were many heterogeneous nucleation regions, the driving force provided by the dislocation during tempering was not enough to promote the M_23_C_6_ phase precipitation. Although 0-II had higher dislocation density, there were few heterogeneous nucleation regions and little precipitation. Moreover, not only did 900-II and 950-II have more precipitation-heterogeneous nucleation regions, but these also had a relatively high dislocation density; hence, the precipitation content after tempering was higher than in other processes.

**Figure 9:**
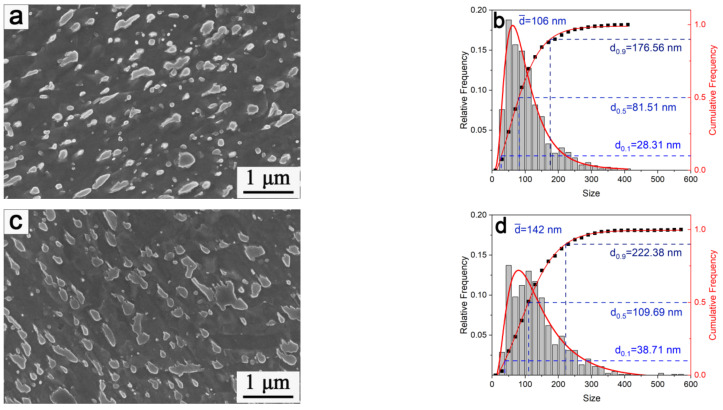
SEM and size distribution of precipitates after aging at 650 °C for 128 h: (**a**,**b**) 900-II; and (**c**,**d**) 0-II.

Comparing the microstructures of 900-II and 0-II after aging for 128 h at 650 °C (Figure 9), the size of the precipitates of 900-II increased from 79 to 106 nm. The size of the 0-II precipitates increased from 112 to 142 nm, and the nano-precipitated phase disappeared. This was because the carbides would undergo Ostwald ripening at 650 °C. The smaller-sized precipitates tend to dissolve in the matrix under the influence of interfacial energy. The larger precipitates continue to coarsen. The increase in the size of the precipitates reduces the precipitation strengthening effect. This promotes crack initiation due to the reduced matching degree with the matrix. Related to 900-II and 0-II, the volume fraction of VC of 900-II was higher than that of 0-II. The volume fraction of M_7_C_3_ was lower than that of 0-II, and the curing rate of VC at 650 °C was much lower than that of M_7_C_3_. Thus, the microstructure stability of 900-II was better than that of 0-II.

### 3.2. Analysis of Mechanical Property

**Figure 10:**
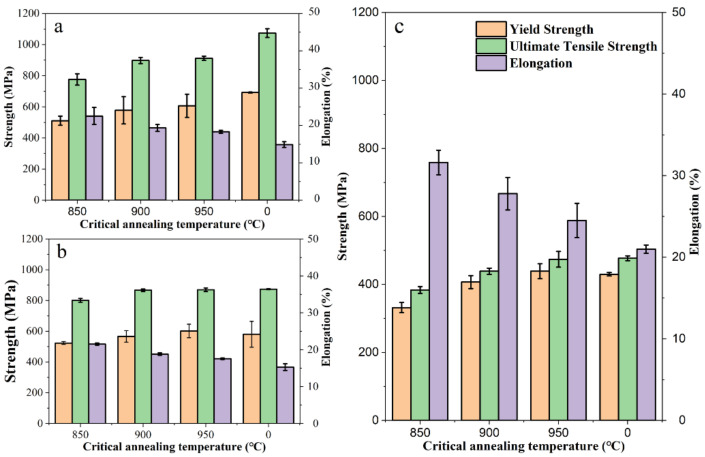
The mechanical properties at (**a**) room temperature; (**b**) room temperature after aging at 650 °C for 128 h; and (**c**) 650 °C.

Figure 10a shows the room temperature mechanical properties of 0-II, 850-II, 900-II, and 950-II samples. The sample 0-II showed the highest yield strengths (YS), UTS and the lowest elongation with values corresponding to 692 MPa, 1074 MPa, and 14.9%, respectively. The ductility of the annealed samples increased while the strength decreased. After intercritical annealing at 850 °C, the YS and UTS decreased to 511 and 776 MPa, while the elongation increased to 22.54%. The strength increased and the plasticity decreased slightly. The products of strength and plasticity are the largest with a UTS of 898 MPa and an elongation of 19.35% at 900 °C intercritical annealing. By stretching at 650 °C (Figure 10c), the strength of all samples decreased and the elongation increased by the action of thermal softening. The UTS of 0-II was only 477 MPa, which was 597 MPa lower than the room temperature strength, and the TEL was only 21%. However, the UTS of 900-II was 439 MPa, the TEL reached 27.8%, and the UST×TEL of 12.2 GPa%.

After aging at 650 °C for 128 h, the UTS of 0-II decreased by 200 MPa compared with that before heat preservation, to only 874 MPa, as shown in Figure 10b. The plasticity had a little change of only 15.29%. The stability of the mechanical properties of the annealed samples was higher than that of the annealed samples. The UTS of the samples annealed at 900 and 950 °C decreased to 867 and 870 MPa while the plasticity decreased to 18.83 and 17.57%, respectively. The 900-II showed the best mechanical property stability.

The high strength obtained at 0-II at room temperature is attributed to the high dislocation density and small substructure size (Table 2), while the low plasticity is due to the precipitation of large-size M_7_C_3_ and M_23_C_6_ phases along the PAGBs and lath boundary (Figure 5g). When the steel is subjected to tensile loads, the deformation coordination between large irregular carbides and matrix becomes poor, and stress concentration and cracks are easy to initiate at grain boundaries which significantly reduces the plasticity of the steel. There are also largely precipitated phases at the interface of 850-II, but mainly spherical VC, which is not easy to produce stress concentration. Meanwhile, the highest plasticity is obtained due to the softening of the matrix. The precipitates in 900-II and 950-II are evenly distributed and become small in size, the precipitate hardening effect to be large, and both the strength and plasticity become relatively good.

At high temperature, the ability of grain boundary sliding is improved due to the effect of thermal activation [32]. Meanwhile, the migration and interaction of dislocation will lead to a decrease in the dislocation density [33]. Therefore, fine grain size and high dislocation density are not conducive to maintain the strength of the metallic material at high temperatures. This explains why the strength of 0-II decreases more than the 900-II and 950-II at 650 °C. Fine precipitates have a more predominant effect by pinning dislocation movement on the tensile property than M_7_C_3_ and M_23_C_6_ at high temperatures. There are also a large number of nano-precipitated phases inside the grains of 0-II (Figure 5g) which lead to similar yield strengths that can be obtained in 0-II and 950-II. In the precipitation phase of 0-II, the content of more thermodynamically stable VC is relatively less, mainly M_7_C_3_, which is easy to coarsen (Table 2). Therefore, after continuous high-temperature exposure, the nanoprecipitate phase disappears and the M_7_C_3_ phase coarsens (Figure 9), resulting in making its yield strength lower than 950-II.

## 4. Conclusions

(1) The intercritical annealing at 850, 900, and 950 °C increased the volume fraction of VC from 2.23 to 3.03, 3.05, and 3.48%, respectively. Meanwhile, annealing at 900 and 950 °C could effectively reduce the size of the precipitates from 112 to 79 and 81 nm.
(2) Increasing the VC volume fraction inhibited the M_7_C_3_ precipitation from 10.01 to 6.63~5.72% during tempering. The M_23_C_6_ phase precipitation was jointly affected by the dislocation density and the nucleation point. The M_23_C_6_ phase would preferentially attach to the VC nucleation at high dislocation density, and the volume fraction of VC was proportional to that of the M_23_C_6_ phase. At 950 °C annealing, the content of M_23_C_6_ increased from 0.15% to 1.02%.
(2) Increasing the annealing temperature, the UTS increased and the TEL decreased. An excellent combination (room temperature: ultimate tensile strength (UTS) of 898 MPa and total elongation (TEL) of 19.35%, 650 °C: UTS of 439 MPa, and TEL of 27.80%) could be obtained when intercritically annealing at 900 °C. Meanwhile, after aging at 650 °C for 128 h, the room temperature UTS and TEL decreased by only 31 MPa and 0.52%, respectively. It is suggested that the H13 steel of higher VC volume fraction has better high-temperature stability from the standpoint of mechanical properties after aging at 650 °C for 128 h.

## Figures and Tables

**Table 1:** The chemical composition of the H13 steel (wt.%).

Element	C	Cr	Mo	Si	V	Mn	P	S
Content (wt.%)	0.385	5.095	1.221	0.972	0.889	0.443	0.017	0.0045
Standard deviation	0.033	0.115	0.139	0.062	0.151	0.113	0.006	0.0015

**Table 2:** Precipitate size, volume fraction, density, lath size, and ρ^GND^ of stage II.

Substructure Size (μm)	ρ^GND^ (nm^−2^)		Precipitate Size (nm)	Volume Fraction (%)			
				Sum	VC	M_7_C_3_	M_23_C_6_
850-II	1.98	0.59 × 10^−3^	113 ± 15.96	9.67	3.03	6.63	0
900-II	1.64	1.31 × 10^−3^	79 ± 2.94	9.82	3.05	6.06	0.71
950-II	1.45	1.82 × 10^−3^	83 ± 7.24	10.23	3.48	5.72	1.02
0-II	1.33	1.98 × 10^−3^	112 ± 21.92	12.40	2.23	10.01	0.15

## Data Availability

Not applicable.
